# Throwing Shoulder Range of Motion and Hamstring Flexibility in Adolescent Baseball Players: A Pilot Study

**DOI:** 10.7759/cureus.46159

**Published:** 2023-09-28

**Authors:** Davong D Phrathep, Jayden Glover, Brandon Valls, Steven Fischer, Jonathan Nguyen, David Boesler

**Affiliations:** 1 Department of Medicine, Lake Erie College of Osteopathic Medicine, Bradenton, USA; 2 Department of Urology, Lake Erie College of Osteopathic Medicine, Jacksonville, USA; 3 Department of Pain Management, Lake Erie College of Osteopathic Medicine, Tampa, USA; 4 Department of Osteopathic Medicine, Lake Erie College of Osteopathic Medicine, Bradenton, USA

**Keywords:** hamstring tightness, pediatric sports medicine, baseball, biomechanics of baseball pitching, shoulder range of motion

## Abstract

Overhead throwing athletes with limited hamstring flexibility should be counseled appropriately regarding their risk for shoulder injury. Baseball players should stretch and prepare their bodies to throw to reduce the compensatory mechanisms that may occur from hamstring stiffness. We aim to identify the association between the contralateral hamstring-popliteal angle and throwing shoulder total range of motion (ROM) in high school baseball players. Fifteen high school varsity baseball players (ages 14-18 years old) volunteered for this observational study. All the athletes played five times a week and played different positions. Bilateral hamstring and throwing shoulder total ROMs were measured using a goniometer by the same examiner. Three examiners determined the maximum ROM by joint end feel for each measurement to ensure quality control. Contralateral hamstring flexibility was measured using the popliteal angle test. These measurements were compared to the throwing shoulder total ROM to determine the presence of an association. The relationship between the contralateral hamstring flexibility and throwing shoulder total ROM was determined using a linear regression analysis. The correlation coefficient (r) was +0.3928, indicating a positive linear relationship. We conclude that higher contralateral hamstring-popliteal angles may cause a compensatory increase in throwing shoulder total ROM. We highlight the role hamstring stiffness may play in shoulder injury development in adolescent baseball players.

## Introduction

Shoulder injuries in the sport of baseball are very common. Previous studies have demonstrated that the significant amount of repetition and stress load on the shoulder throughout the whole overhead throwing motion in baseball can lead to shoulder injuries [1]. Specifically, adolescent baseball players can be at increased risk of developing injuries due to many different risk factors including increased weight, decreased height, lifting weights during the season, playing baseball in multiple leagues, arm fatigue, and excessive pitch counts. A prospective study of youth baseball pitchers over two seasons showed that 32% of the athletes complained of shoulder pain [2]. Another prospective study showed that young baseball pitchers are at a 5% risk within 10 years of developing serious injuries including elbow surgery, shoulder surgery, or retirement from baseball due to a throwing injury [3]. The risk of injury from playing other positions in baseball without pitching in youth baseball players has not been well studied. We designed a study including an entire baseball team to determine risk factors that can potentially result in arm injuries in baseball players of all positions.

The overhead throwing motion in baseball is very complex (different shoulder angles, release points, and arm speed), and the biomechanics of overhead throwing are not exclusively confined to the upper extremities. The throwing motion involves a transfer of energy throughout many different parts of the body, including the trunk [4]. Therefore, large vector forces are not only confined to the shoulder alone. The general mechanism of the overhead throwing motion can be described as the sequential acceleration and deceleration of the athlete's body. This mechanism can be described in six phases: windup, stride (early cocking), late cocking, acceleration, deceleration, and follow-through [5]. As it is known, the hamstring muscles are significantly involved in the stride and deceleration phases, specifically during stride length and contralateral lead leg extension [5]. Overall, tight hamstring muscles can shorten a player's stride and decrease the lead leg's ability to extend, which may decrease a player's force potential and ability to decelerate properly. This complex overhead throwing motion and restrictions in range of motion (ROM) at the hamstring muscles may cause the body to compensate and place additional stress on the throwing shoulder, leading to a higher likelihood of injury. However, the relationship specifically between throwing shoulder total ROM and lead leg hamstring ROM in baseball players has not been well described. However, it has been hypothesized that forces at the throwing shoulder may be greater in an athlete who is compensating for injuries or ROM restrictions at the joints at some distance from the shoulder (e.g., lower back, hip, and ankle) [6]. Although hamstring ROM limitations could result in greater demands on the shoulder in an attempt to achieve maximum efficiency in the throwing motion, we are unaware of any data characterizing differences in contralateral hamstring flexibility and throwing shoulder total ROM in adolescent baseball players. Therefore, the purpose of our study was to determine if differences existed in hamstring and shoulder ROMs in high school baseball players.

## Materials and methods

Participants

A total of 15 high school varsity baseball players (ages 14-18 years old) volunteered for this study. Inclusion criteria included high school-aged male baseball players on the varsity team. Exclusion criteria included athletes with current injuries such as leg or shoulder pain. The 15 baseball players used for the study all played a variety of positions (i.e., infield, outfield, catcher, or pitcher). Four players were left-handed, and 11 players were right-handed. All athletes practice or play roughly five times a week; however, no data was collected regarding throwing frequency. Prior to the conduction of this study, the study received institutional review board approval and obtained informed parental consent for those under 18 years and informed consent from those over 18 years.

Range of motion measurements

Bilateral hamstring and shoulder ROMs were measured for all participants. Hamstring ROM was measured in the supine position for both dominant and nondominant legs. Shoulder ROM was measured in the supine position for both dominant and nondominant shoulders. All measurements were taken by three of the six examiners, while the other three examiners were holding the player in position. Measurements were taken using a goniometer. It has been shown that passive ROM goniometric assessment is the gold standard and reliable when used by the same examiner. Maximum ROM was determined by joint end feel and by using the same three examiners to determine when joint end feel was reached.

Straight leg hamstring ROM was measured with participants lying supine on the table. The leg was then flexed in anatomic neutral until the knee joint end feel was determined while ensuring no knee flexion occurs. The goniometer's axis was aligned on the greater trochanter, with the stable arm aligned on the torso and the moveable arm aligned with the midshaft of the femur. This procedure was repeated on the opposite side.

Shoulder external rotation (ER) and internal rotation (IR) ROM were measured with the player lying supine on the table and legs straight. The shoulder was abducted to 90 degrees and the elbow was flexed to 90 degrees, ensuring that the humerus was parallel to the ground. It is essential that the player's elbow is off the table to prevent the table from limiting external and internal rotation. To induce external rotation, the player's arm was passively moved from the neutral position of 90 degrees shoulder abduction and 90 degrees shoulder flexion to moving the forearm posteriorly until the end feel was determined. The examiner responsible for moving the player into position also provided shoulder stabilization with the opposite hand. Maximum ER was measured with the goniometer's axis on the olecranon process of the ulna, the stable arm perpendicular to the floor and the moveable arm aligned with the ulnar styloid process. IR was achieved by moving the player's arm from the neutral position in 90 degrees abduction and 90 degrees flexion to moving the forearm anteriorly until the end feel was determined. The examiner moving the player into position also provided stabilization to the shoulder by placing a hand on the anterior aspect of the shoulder. Maximum IR was measured by placing the goniometer's axis on the olecranon process of the ulna, the stable arm perpendicular to the floor and the moveable arm aligned with the ulnar styloid process. This procedure involved glenohumeral rotation being measured with the patient in the supine position, the shoulder abducted 90 degrees in the plane of the body, and the scapula stabilized against the examination table by downward pressure applied by the examiner to the anterior aspect of the shoulder. In order to compare dominant to nondominant glenohumeral IR and ER, total ROM was also assessed by adding the ER on both sides and the same for IR. Total ROM measurements were collected bilaterally. Total ROM was then compared to the contralateral hamstring flexibility to determine if there is a correlation between shoulder ROM and hamstring flexibility.

All players completed an injury participation questionnaire. The questionnaire included past medical history, prior arm injuries, prior leg injuries, current arm injuries, current leg injuries, and throwing arm. Any injury was defined as a sports-related injury that has occurred within the player's baseball career. The injury report from the players was a self-reported recollection of injuries from the athlete's past playing experience.

## Results

On the medical history questionnaire, six players reported a history of injury, with four being upper extremity injuries and two being lower extremity injuries. Nine players reported no history of injury. One player reported both a history of an upper extremity and lower extremity injury. There were four left-handed players and 11 right-handed players. The measurements for hamstring flexibility, shoulder internal rotation, and shoulder external rotation are shown in Table 1.

**Table 1:** Hamstring flexibility, shoulder internal rotation, and shoulder external rotation measurements

Player number	Handedness	History of injury	Right hamstring (degrees)	Left hamstring (degrees)	Right shoulder internal rotation (degrees)	Left shoulder internal rotation (degrees)	Right shoulder external rotation (degrees)	Left shoulder external rotation (degrees)
1	Left	No	52	55	76	61	107	122
2	Right	No	54	40	82	74	101	91
3	Left	No	51	48	84	70	88	110
4	Right	Strained oblique	31	47	88	82	101	87
5	Right	Strained hip flexor	43	37	81	69	96	92
6	Left	Strained forearm	31	37	80	88	81	64
7	Right	No	34	33	62	60	88	96
8	Right	Broken right elbow and left strained hamstring	20	40	80	78	120	93
9	Right	Torn labrum	31	33	59	108	91	64
10	Right	No	47	52	51	64	104	86
11	Right	No	51	38	68	84	92	81
12	Right	No	44	25	65	82	108	102
13	Left	No	29	30	65	72	84	82
14	Right	Torn labrum	61	69	70	86	111	70
15	Right	No	19	20	60	64	110	90

The measurements for contralateral hamstring flexibility and total throwing shoulder ROM are shown in Table 2.

**Table 2:** Hamstring flexibility and throwing shoulder total range of motion

Player number	Contralateral hamstring flexibility (degrees)	Shoulder total range of motion (degrees)
1	52	183
2	51	180
3	31	152
4	29	154
5	40	183
6	47	189
7	37	177
8	33	150
9	40	200
10	33	150
11	52	155
12	38	160
13	25	173
14	69	181
15	20	170

**Figure 1:**
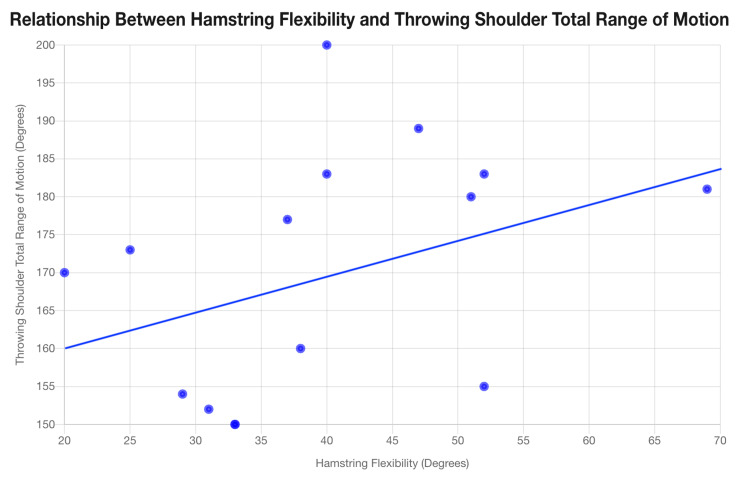
Relationship between hamstring flexibility and throwing shoulder total range of motion

The relationship between the contralateral hamstring flexibility and total throwing shoulder ROM was determined using a linear regression analysis. The correlation coefficient (r) was +0.3928, indicating a positive linear relationship (Figure 1).

We conclude that higher contralateral hamstring-popliteal angles may have a potential association with a compensatory increase in total throwing shoulder ROM.

## Discussion

**Figure 2:**
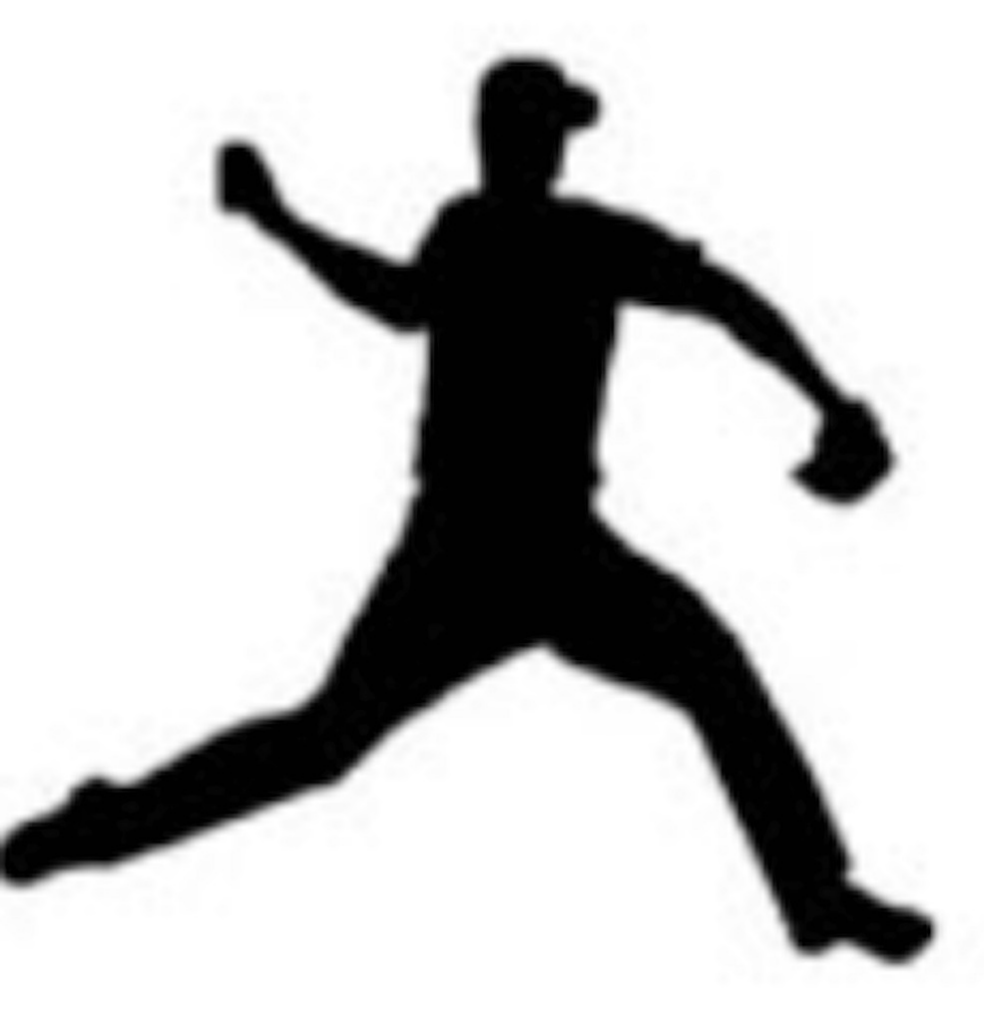
Stride position of the throwing motion Note: Image sourced from "Pitching mechanics, revisited," by Calabrese GJ, 2013, Int J Sports Phys Ther, 8(5):652-60 [5] under Creative Commons license CC-BY-NC-SA.

Restricted hamstring range of motion may place additional stress on the throwing shoulder and increase the risk of injury. However, the relationship between the contralateral hamstring flexibility and throwing shoulder total ROM in adolescent baseball players is unknown. One of the most important and common components that every different position player (e.g., pitcher, infielder, catcher, and outfielder) in baseball needs to execute a proper overhead throwing motion is the "stride position" [5]. The stride position allows for proper positioning of the trunk and lower extremity to transfer energy to the upper extremity by increasing the time and distance that the trunk can rotate (Figure 2).

A short stride would decrease the potential force produced due to reduced trunk rotation time and distance. An important limitation to stride length is hamstring flexibility in the lead leg during the throwing motion [5]. To complete the stride to foot contact position, the knee and hip extensors of the lead leg operate in an eccentric manner while the tensor fascia latae, gluteus medius, and hamstring muscles control internal rotation and posterior tilt of the hips. Moreover, the gluteus maximus works with the semitendinosus and semimembranosus muscles to extend the hip. The gluteus maximus also works in conjunction with the iliopsoas, piriformis, and obturator muscles to externally rotate the hip as the upper extremity prepares to release the baseball [7].

**Figure 3:**
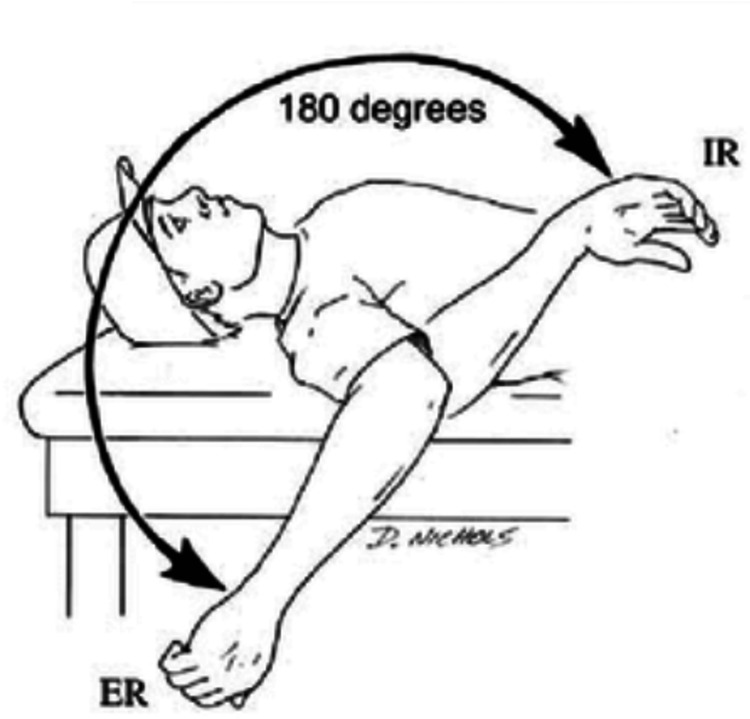
Combination of external rotation and internal rotation equals total motion of 180 degrees ER: external rotation, IR: internal rotation Note: Image sourced from "Microinstability of the shoulder in the overhead athlete," by Reinold MM and Curtis AS, 2013, Int J Sports Phys Ther, 8:601-16 [8] under Creative Commons license CC-BY-NC-SA.

Normally, the internal rotation of the shoulder is 70-90 degrees, the external rotation of the shoulder is 90 degrees, and the total range of motion of the shoulder is 180 degrees (Figure 3) [8].

**Figure 4:**
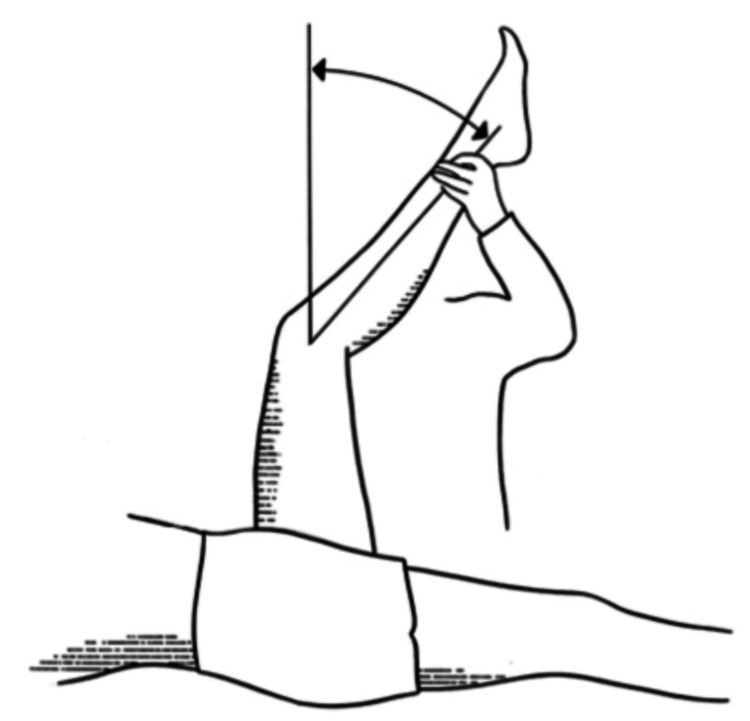
Popliteal angle test Note: Image sourced from "Interrater reliability for unilateral and bilateral tests to measure the popliteal angle in children and youth with cerebral palsy," by Cloodt E, Krasny J, Jozwiak M, and Rodby-Bousquet E, 2021, BMC Musculoskelet Disord, 22:275. 10.1186/s12891-021-04135-6 [9] under Creative Commons Attribution License 4.0.

In baseball players, the external rotation of the shoulder tends to be greater and the internal rotation of the shoulder tends to be smaller, but nonetheless, the total range of motion tends to remain approximately 180 degrees. In this study, we used the popliteal angle test to determine hamstring flexibility, with normal values being less than 30 degrees (Figure 4) [9].

The study measurements were taken prior to the beginning of baseball practice in the afternoon when the players had not stretched yet. The basis of this study was to calculate lead leg hamstring flexibility and compare it to the player's dominant throwing shoulder total range of motion. More specifically, the purpose of this study was to determine the association(s) between the contralateral hamstring-popliteal angle and total throwing shoulder ROM in high school baseball players. Thirteen players demonstrated limited lead leg hamstring flexibility with values being greater than 30 degrees. Additionally, all players with a higher-than-normal total range of motion in the throwing shoulder demonstrated reduced hamstring flexibility. Our study concluded that there was a moderate association between higher contralateral hamstring-popliteal angles and higher total throwing shoulder ROM. We found that reduced hamstring flexibility could cause excessive throwing shoulder total range of motion, which serves as a potential risk factor for injury in adolescent baseball players.

It is important to note that six players recorded previous injuries. Player 4 had a history of a partial oblique tear, player 5 had a history of a strained hip flexor, player 6 had a history of a strained throwing forearm, player 8 had a history of a broken throwing dominant elbow and contralateral hamstring strain, and player 9 and player 14 had both a history of a torn labrum. It seems that players with a history of injuries had a higher throwing shoulder range of motion in comparison to those without injuries. One interesting finding that is important to note is that we had one player who had a previous past history of a left hamstring strain and on examination demonstrated the highest total shoulder range of motion of 200 degrees. Because of our findings, we aim to outline the mechanism by which inflexible lead leg hamstring musculature can increase the risk of throwing shoulder injury due to excessive total range of motion as a compensatory mechanism in throwing athletes.

Hamstring strains are the most prevalent and serious non-contact occurring injury in sports [10]. A hamstring strain, also known as a pulled hamstring, can occur when the semitendinosus, semimembranosus, and/or biceps femoris get overstretched and begin to tear. This leads to reduced hamstring flexibility and compensatory mechanisms to reduce pain with any movement that over-stretches the musculature. Hamstring strains can range from mild to severe but nonetheless can increase the risk of shoulder injury in overhead throwing athletes. Since the rate of upper extremity injury is high in high school baseball players, it was hypothesized in our study that this could be due to tightness and instability of the lower extremity, specifically focusing on the flexibility of the lead leg hamstring muscles. Weak hamstring muscles can lead to anterior pelvic tilt, excessive lumbar lordosis, and low back pain, resulting in increased tension in the thoracolumbar fascia [11]. In recent studies on high school baseball players, hamstring tightness on the non-throwing arm side was shown to be a potential risk factor for low back pain [12]. Because the latissimus dorsi connects the spine to the humerus, tightness in this muscle can decrease glenohumeral joint function and cause inflammation in the fascia connecting the latissimus dorsi to the thoracic and lumbar spine [13]. Through this, a tight latissimus dorsi has been shown to be a cause of chronic shoulder pain and back pain [14]. The compensatory increase in tension and contraction of the latissimus dorsi places a larger stress load on the shoulders due to its insertion on the humerus, which can be exacerbated by hamstring inflexibility. Chronic shoulder pain has been reported to improve with therapeutic thoracolumbar fascia release [14]. The connection between the hamstrings, thoracolumbar fascia, and glenohumeral joint demonstrates how a tense thoracolumbar fascia and latissimus dorsi secondary to hamstring strain and inflexibility can be associated with shoulder pain and increased risk for shoulder injury. Our study suggests that reduced hamstring flexibility may be a potential risk factor for increased upper extremity load, resulting in excessive throwing shoulder total range of motion and injury. Overhead throwing athletes with limited hamstring flexibility should be counseled appropriately regarding their risk for shoulder injury. Baseball players should stretch and prepare their bodies to properly throw to reduce the compensatory mechanisms that may occur from hamstring inflexibility.

While the study found a correlation between higher contralateral hamstring flexibility and increased throwing shoulder range of motion, it cannot establish causation. This is a limitation of the study because other factors such as training routines, muscle imbalances, or biomechanics could also contribute to the observed relationship. Instead, it is important to note that this study highlights hamstring flexibility as a potential factor that may affect throwing shoulder total range of motion, which was the goal of this study. Additionally, another limitation of this study is that the small sample size may affect the generalizability and reliability of the study's findings. In the future, we urge further research regarding hamstring flexibility and throwing shoulder total range of motion in larger sample sizes to improve the generalizability and reliability of our findings. Lastly, the study did not have a control group of players with normal hamstring flexibility. Without a control group, it was challenging to compare the results and draw a more robust conclusion about the association between hamstring flexibility and throwing shoulder total range of motion. This being said, we urge further research to be done to enhance our findings in larger studies. Our pilot study provides a foundation for the future of research regarding the association between contralateral hamstring flexibility and throwing shoulder total range of motion in adolescent baseball players.

## Conclusions

Due to shoulder injuries being common in baseball players, it is essential to explore all components of the overhead throwing motion. Studying the relationship between the hamstring muscles and the dominant throwing shoulder in overhead throwing athletes can bring new insight into how to prevent shoulder injuries in the future. Further research that includes more data and larger sample sizes associated with the development of overhead throwing shoulder injuries related to lead leg hamstring inflexibility is necessary. Our study highlights the relationship between lead leg hamstring ROM and throwing shoulder total ROM in a high school baseball team of 15 players. We concluded a potential correlation between higher popliteal angles and higher throwing shoulder total range of motion. Due to the important role hamstring muscles play in achieving the stride position of the throwing motion, we present the novel finding that hamstring inflexibility may play a role in shoulder injury development due to excessive compensatory increases in throwing shoulder total range of motion in adolescent baseball players.
